# Altered regulation of Ia afferent input during voluntary contraction in humans with spinal cord injury

**DOI:** 10.7554/eLife.80089

**Published:** 2022-09-07

**Authors:** Bing Chen, Monica A Perez

**Affiliations:** 1 Shirley Ryan AbilityLab, Northwestern University, and Edward Hines Jr., VA Medical Center Chicago United States; Icahn School of Medicine at Mount Sinai United States; Icahn School of Medicine at Mount Sinai United States

**Keywords:** H-reflex, motor neuron, presynaptic inihibition, None

## Abstract

Sensory input converging on the spinal cord contributes to the control of movement. Although sensory pathways reorganize following spinal cord injury (SCI), the extent to which sensory input from Ia afferents is regulated during voluntary contraction after the injury remains largely unknown. To address this question, the soleus H-reflex and conditioning of the H-reflex by stimulating homonymous [depression of the soleus H-reflex evoked by common peroneal nerve (CPN) stimulation, D1 inhibition] and heteronymous (d), [monosynaptic Ia facilitation of the soleus H-reflex evoked by femoral nerve stimulation (FN facilitation)] nerves were tested at rest, and during tonic voluntary contraction in humans with and without chronic incomplete SCI. The soleus H-reflex size increased in both groups during voluntary contraction compared with rest, but to a lesser extent in SCI participants. Compared with rest, the D1 inhibition decreased during voluntary contraction in controls but it was still present in SCI participants. Further, the FN facilitation increased in controls but remained unchanged in SCI participants during voluntary contraction compared with rest. Changes in the D1 inhibition and FN facilitation were correlated with changes in the H-reflex during voluntary contraction, suggesting an association between outcomes. These findings provide the first demonstration that the regulation of Ia afferent input from homonymous and heteronymous nerves is altered during voluntary contraction in humans with SCI, resulting in lesser facilitatory effect on motor neurons.

## Introduction

Anatomical and physiological studies have shown that sensory input to motor neurons is altered following spinal cord injury (SCI) (DAmico et al., 2014). For example, lesions of descending motor tracts in animals result in aberrant sprouting of primary afferents, leading to symptoms of hyperreflexia (Murray and Goldberger, 1974; Wong et al., 2000), and prolonged excitatory postsynaptic potentials (EPSPs) are observed in motor neurons in response to brief sensory stimulation (Baker and Chandler, 1987). In agreement, humans with SCI show prolonged depolarization of motor neurons in response to stimulation of sensory nerves (Norton et al., 2008), exaggerated stretch reflexes (Chen et al., 2020), and decreased transmission in spinal inhibitory pathways compared with uninjured controls (Jones and Yang, 1994; Crone et al., 2003; DeForest et al., 2020). The functional consequences of this altered sensory input conveying to motor neurons following SCI remain largely unknown.

Primary afferent fibers (Ia) are rapidly conducting sensory fibers that originate from muscle spindle primary endings, which constantly monitors the rate at which a muscle stretch changes (Matthews, 1972). Ia afferent fibers bifurcate on entering the spinal cord and run several segments in both rostral and caudal directions in the dorsal columns and make contact with motor neurons (Pierrot-Deseilligny and Burke, 2012). Different mechanisms can contribute to regulate Ia afferent input conveying to motor neurons. For decades, it was thought that sensory regulation was accomplished in part through axoaxonic contacts at the terminal of Ia sensory axons from GABAergic neurons that receive innervation from the brain and spinal cord through presynaptic inhibition (Frank and Fuortes, 1957; Eccles et al., 1961; Eccles et al., 1962; Rudomin and Schmidt, 1999). However, recent evidence in animal and humans suggested that facilitation of Ia-mediated EPSPs in motor neurons likely occurs when axon nodes of Ranvier are depolarized from activation of nodal GABA_A_ receptors, which contributes to reduce branch point failure in Ia afferent fibers (Hari et al., 2021; Metz et al., 2021). GABAergic neurons known to innervate terminals of Ia afferent fibers in the spinal cord also innervate nodes of Ranvier contributing to prevent failure of sodium spike transmission at branch points, which can facilitate sensory transmission and reflexes (Hari et al., 2021). Furthermore, in humans, cutaneous, proprioceptive, and corticospinal pathways can facilitate rather than inhibit Ia afferents facilitating the propagation of action potentials to motor neurons (Metz et al., 2021). Thus, GABAergic networks can have both facilitatory and inhibitory actions on afferent transmission within the spinal cord at different sites within Ia afferent fibers (Metz et al., 2022).

A critical question is how Ia afferent transmission is regulated during voluntary contraction after SCI. In uninjured humans, evidence showed that Ia afferent transmission decreases at the onset of a voluntary contraction (Hultborn et al., 1987b; Nielsen and Kagamihara, 1993) and during tonic contractions that last for 1–2 min (Meunier and Pierrot-Deseilligny, 1989; Nielsen and Kagamihara, 1993) compared to rest and it changes according to the task requirements (Capaday and Stein, 1986; Crone and Nielsen, 1989). Following SCI, descending motor pathways converging onto GABAergic interneurons thought to contribute to regulate Ia afferent transmission (Jankowska and Edgley, 2006) are likely altered. Indeed, humans with SCI show lesser corticospinal (Davey et al., 1998; Bunday et al., 2014) and H-reflex (Yang et al., 1991; Phadke et al., 2010) modulation during voluntary behaviors compared with control participants. We hypothesized that during voluntary contraction, Ia afferent input exerts a lesser facilitatory effect on motor neurons in SCI compared with control participants.

**Figure 1.**
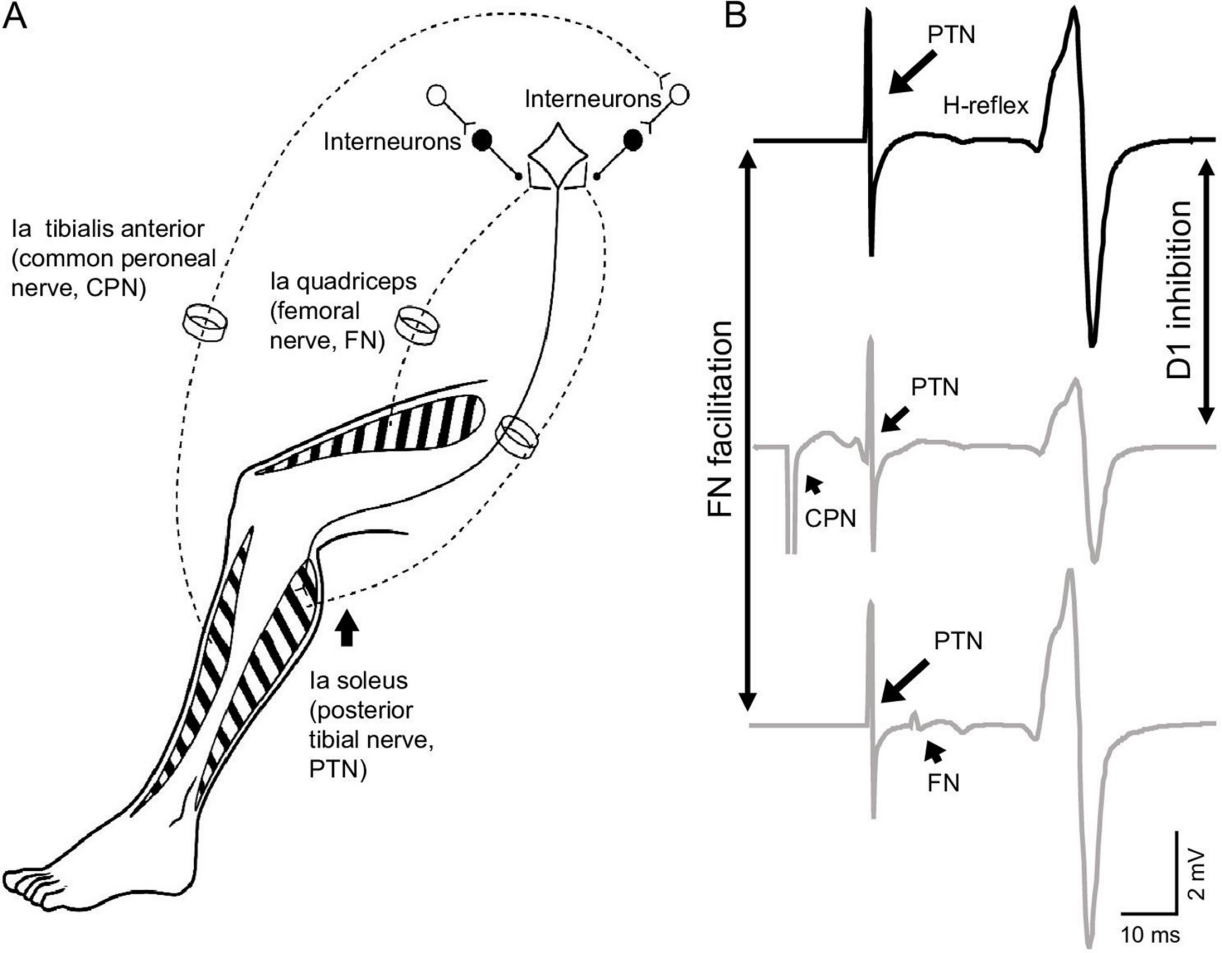
Experimental setup. (**A**) Schematic representation of afferent fibers and motor neurons stimulated during our procedures. The soleus reflex was evoked by electrical stimulation of Ia afferents on the posterior tibial nerve (PTN). We assessed Ia afferent input to motor neurons by measuring the depression of the soleus H-reflex evoked by stimulating Ia afferents on the common peroneal nerve (CPN; referred as ‘D1 inhibition’) and the monosynaptic Ia facilitation of the soleus H-reflex evoked by stimulating Ia afferents on femoral nerve (referred as ‘FN facilitation’) at rest, and during tonic voluntary contraction. (**B**) Representative traces showing the soleus H-reflex evoked by PNT stimulation, the D1 inhibition evoked by stimulation of the CPN preceding the PTN at a conditioning-test interval of 15 ms, and the FN facilitation evoked by stimulation of the FN after the PTN at a conditioning-test interval of –8 ms (negative value of the interval indicates that the stimuli to the PTN precedes the FN stimuli).

To test this hypothesis, the soleus H-reflex and conditioning of the H-reflex by stimulating homonymous and heteronymous nerves by measuring the depression of the soleus H-reflex evoked by common peroneal nerve (CPN) stimulation [D1 inhibition] and the monosynaptic Ia facilitation of the soleus H-reflex evoked by femoral nerve (FN) stimulation [FN facilitation] were tested at rest and during 30% of tonic maximal voluntary contraction (MVC). The position of the ankle joint was maintained constant across conditions to standardize the possible effect of other inputs into the testing procedures (Figure 1).

## Results

### EMG

**Figure 2.**
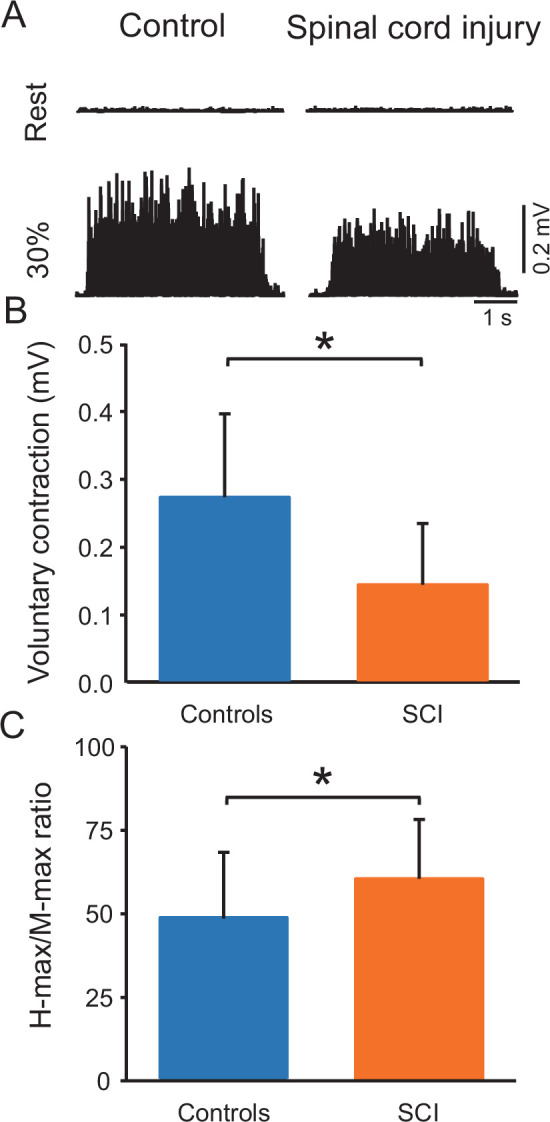
Voluntary contraction and maximal H-reflex and maximal motor response (M-max) ratio (H-max/M-max ratio). (**A**) Electromyographic (EMG) traces tested at rest and during 30% of maximal voluntary contraction (MVC) with the soleus muscle in a control and in a spinal cord injury (SCI) participant. (**B**) Bar graph shows the MVC group data. The abscissa shows the groups tested (controls = blue bar, SCI = orange bar) and the ordinate shows the MVC (in millivolts). (**C**) Bar graph shows the H-max/M-max ratio group data. The abscissa shows the groups tested (controls = blue bar, SCI = orange bar) and the ordinate shows the H-max/M-max ratio. *p<0.05, one-way ANOVA with Holm-Sidak post-hoc analysis. n = 20 per group, error bars show standard diviation (SD).

Figure 2A shows raw rectified EMG data in the soleus muscle in representative control and SCI participants at rest and during 30% of MVC. One-way ANOVA showed an effect of GROUP on MVCs (controls = 0.3 ± 0.1 mV and SCI = 0.1 ± 0.1 mV, p<0.001; F_1,38_=13.7, p=0.001, η^2^p=0.3; Figure 2B) and the maximal motor response (M-max) (controls = 13.2 ± 3.3 mV and SCI = 10.3 ± 4.8 mV; F_1,38_=5.1, p=0.03, η^2^p=0.2) but not the maximal H-reflex (H-max) (controls = 6.2 ± 2.7 mV and SCI = 6.5 ± 4.0 mV, F_1,38_=0.06, p=0.8) in the soleus muscle. We also found a group effect on H-max/M-max ratio (controls = 48.5% ± 19.7%, SCI = 62.0 ± 19.1%, p=0.03, η^2^p=0.2, Figure 2C). Note that there was no difference in the activation of the tibialis anterior muscle between controls and SCI participants during 30% of plantarflexion MVC (controls = 7.3% ± 3.6% of tibialis anterior MVC and SCI = 9.2% ± 10.6% of tibialis anterior MVC, p=0.2).

### Soleus H-reflex

**Figure 3.**
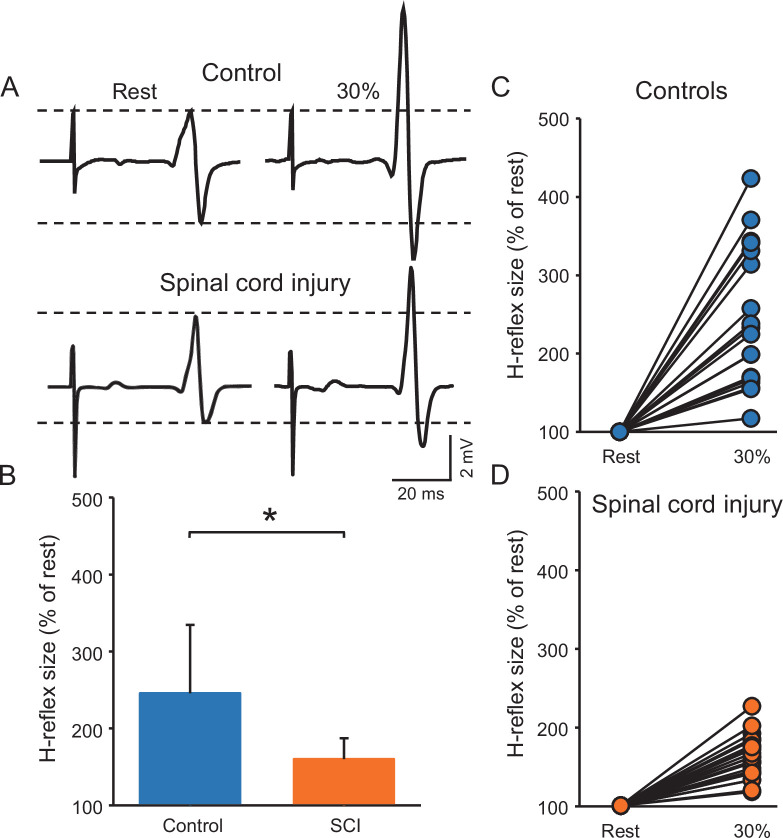
Soleus H-reflex. (**A**) Representative EMG traces showing the soleus H-reflex tested at rest and during 30% of MVC in a control and in a SCI participant. (**B**) Graph shows the group H-reflex data. The abscissa shows the groups tested (controls = blue bar, SCI = orange bar) and the ordinate shows the H-reflex size during 30% of MVC expressed as a % of the H-reflex size at rest. Graphs show individual H-reflex data in controls (**C**) and SCI (**D**) participants. The abscissa shows the conditions tested (rest, 30% of MVC) and the ordinate shows the H-reflex size during 30% of MVC expressed as a % of the H-reflex size at rest. *p<0.05, repeated measures ANOVA with Holm-Sidak post-hoc analysis. n = 20 per group, error bars show SD.

Figure 3A illustrates raw traces showing the soleus H-reflex in a control and a SCI participant. Note that the H-reflex increased in both participants during voluntary contraction compared with rest but to a lesser extent in the individual with SCI. Repeated measures ANOVA showed an effect of GROUP (F_1,38_=15.6, p<0.001, η^2^p=0.3), CONTRACTION (F_1,28_=100.3, p<0.001, η^2^p=0.7) and in their interaction (F_1,38_=15.6, p<0.001, η^2^p=0.3) on the H-reflex size. Post hoc analysis showed that the H-reflex was larger during 30% of MVC (controls = 245.6% ± 88.7%, p<0.001; SCI = 163.2 ± 23.2%, p<0.001) compared to rest in both groups. Additionally, the H-reflex size increased to a lesser extent in SCI compared to control subjects at 30% of MVC (p<0.001, Figure 3B, C and D). Note that the soleus H-reflex was tested during 30% of MVC (controls = 29.8% ± 7.1% of MVC and SCI = 33.2% ± 4.0% of MVC, p=0.2) into plantarflexion in both groups.

We conducted an additional control experiment where we matched absolute EMG level across groups during 30% of MVC by asking control participants to perform the similar EMG activity as SCI participants. Repeated measures ANOVA showed an effect of GROUP (F_1,18_=6.0, p=0.02, η^2^p=0.3), CONTRACTION (F_1,18_=52.4, p<0.001, η^2^p=0.7), and in their interaction (F_1,18_=6.1, p=0.02, η^2^p=0.3) on the H-reflex size. Post hoc analysis showed that the H-reflex was larger during 30% of MVC (controls = 218.9% ± 73.7%, p<0.001; SCI = 158.12 ± 23.1%, p<0.001) compared to rest in both groups. Note that the increases in H-reflex size were lesser in SCI compared with control at 30% of MVC (p=0.02).

### D1 inhibition

**Figure 4.**
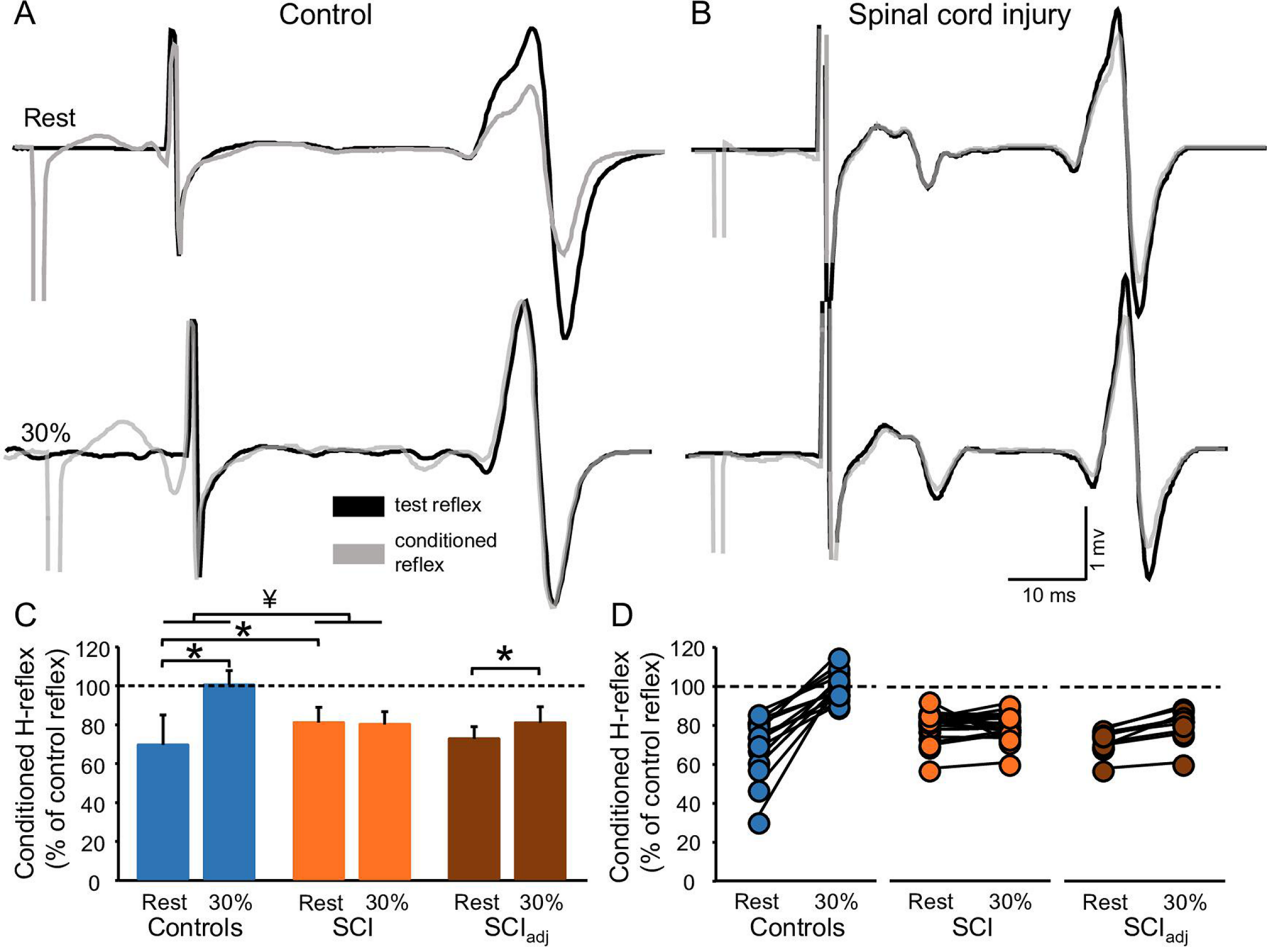
D1 inhibition. Representative traces showing the H-reflex (control reflex, in black) and the H-reflex conditioned by common peroneal nerve (CPN) stimulation (conditioned H-reflex, in gray) tested at rest and during 30% of MVC in a control (**A**) and in a SCI (**B**) participant. Note that during 30% of MVC we show the test H-reflex adjusted. The bar graph shows the conditioned H-reflex normalized to the control H-reflex in both groups (**C**). The abscissa shows the groups tested at rest and during 30% of MVC (controls = blue bars, SCI = orange bars, SCI_adj_ = brown bars). Note that here the SCI_adj_ condition refers to testing of the D1 inhibition_adj_. The ordinate shows the size of conditioned H-reflex expressed as a % of the control H-reflex (use to assess the D1 inhibition). Data from individual subjects (**D**) showing the conditioned H-reflex normalized to the control H-reflex in all groups tested (controls = blue circles, SCI = orange circles, SCI_adj_ = brown circles). *p<0.05, repeated measures ANOVA with Holm-Sidak post-hoc analysis. Controls n = 14, SCI n = 20, SCI_adj_ n = 10, error bars show SD.

Figure 4A and B illustrates raw traces showing the D1 inhibition measured in a representative control and SCI participant. Compared with rest, it shows that the D1 inhibition was abolished during 30% of MVC in the control participant while the D1 inhibition remained unchanged in the SCI participant. Repeated measures ANOVA showed an effect of GROUP (F_1,32_=6.4, p=0.01, η^2^p=0.1), CONTRACTION (F_1,32_=36.6, p<0.001, η^2^p=0.6), and in their interaction (F_1,32_=20.6, p<0.001, η^2^p=0.7) on the D1 inhibition. Post hoc analysis showed that the D1 inhibition decreased during 30% of MVC in controls (rest = 69.6% ± 15.4%, 30% of MVC = 100.5% ± 7.2%, p<0.001) but not in SCI (rest = 81.2% ± 7.8%, 30% of MVC = 80.2% ± 6.6%, p=0.5; Figure 4C) participants. Because D1 inhibition at rest is decreased in SCI (81.2% ± 7.8%) compared with control (69.6% ± 15.4%, p=0.02) participants, we tested the D1 inhibition in a subgroup of SCI participants (n=10) by adjusting to magnitude of the D1 inhibition to match the values obtained in control subjects (D1 inhibition_adj_, see Materials and methods). Here, we found that the D1 inhibition_adj_ decreased during 30% of MVC compared with rest in SCI participants (rest = 72.9% ± 6.1%, 30% of MVC = 81.1% ± 8.2%, p<0.001; Figure 4C–D) but to a lesser extent than controls.

### FN facilitation

**Figure 5.**
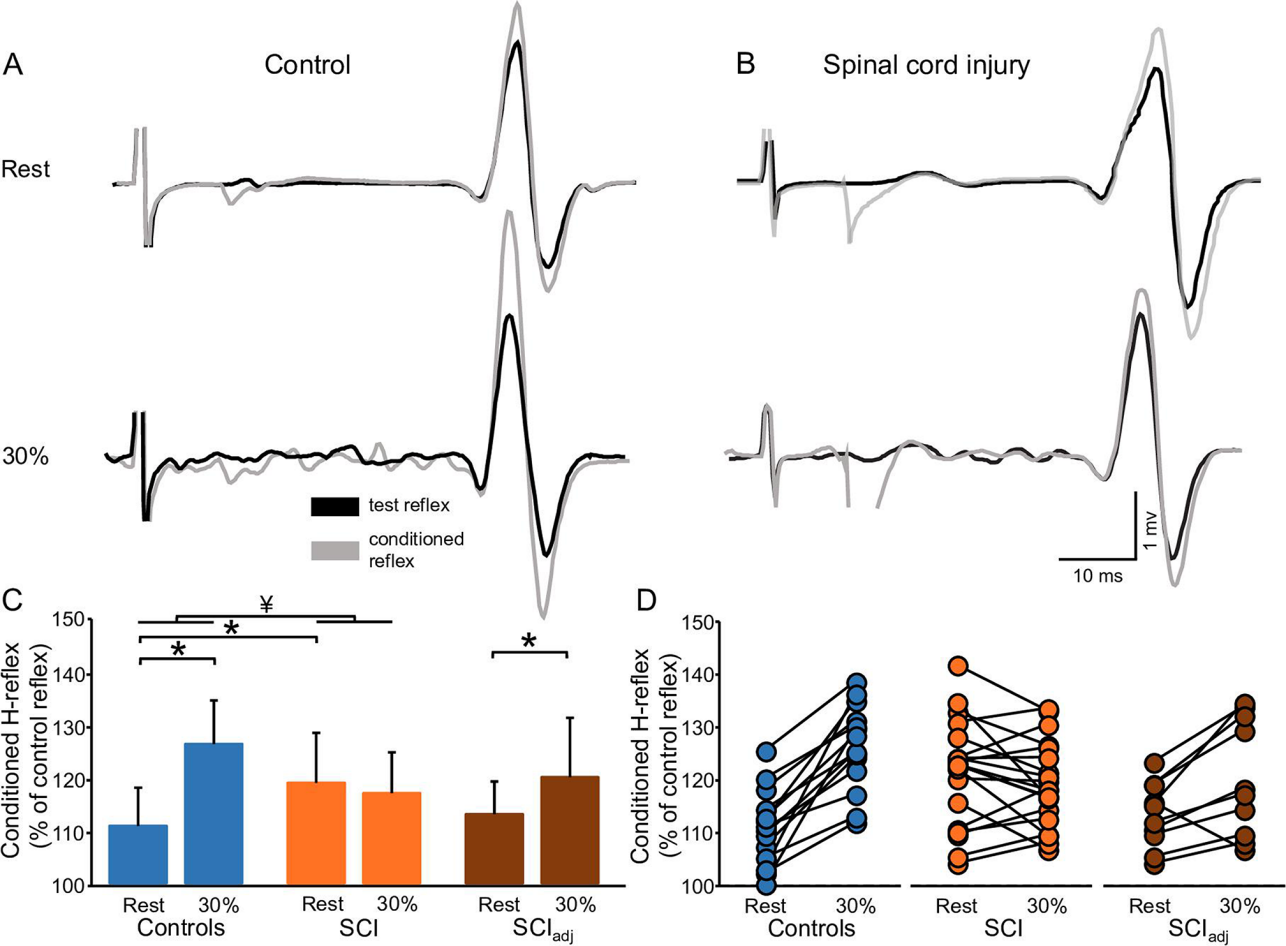
FN facilitation. Representative traces showing the H-reflex (control reflex, in black) and the H-reflex conditioned by FN stimulation (conditioned H-reflex, in gray) tested at rest and during 30% of MVC in a control (**A**) and in a SCI (**B**) participant. Note that during 30% of MVC we show the test H-reflex adjusted. The bar graph shows the conditioned H-reflex normalized to control H-reflex in both groups (**C**). The abscissa shows the groups tested at rest and during 30% of MVC (controls = blue bars, SCI = orange bars, SCI_adj_ = brown bars). Note that here the SCI_adj_ condition refers to the testing of the FN facilitation_adj_. The ordinate shows the size of the conditioned H-reflex expressed as a % of the control H-reflex (used to assess the FN facilitation). Data from individual subjects (**D**) showing the conditioned H-reflex normalized to the control H-reflex in all groups tested (controls = blue circles, SCI = orange circles, SCI_adj_ = brown circles). *p<0.05, repeated measures ANOVA with Holm-Sidak post-hoc analysis. Controls n = 14, SCI n = 20, SCI_adj_ n = 10, error bars show SD.

Figure 5A and B illustrates raw traces showing the FN facilitation measured in a control and in an SCI participant. We found that the FN facilitation increased in the control but not in the SCI participant during 30% of MVC compared with rest. Repeated measures ANOVA showed an effect of GROUP (F_1,32_=4.8, p=0.04, η^2^p=0.1), CONTRACTION (F_1,32_=30.8, p<0.001, η^2^p=0.5), and in their interaction (F_1,30_=51.5, p<0.001, η^2^p=0.6) on the FN facilitation. Post hoc analysis showed that the FN facilitation increased during 30% of MVC in controls (rest = 111.0% ± 7.1%, 30% of MVC = 126.3% ± 8.2%, p<0.001) but not in SCI (rest = 119.2% ± 9.3%, 30% of MVC = 117.2% ± 7.6%, p=0.16; Figure 5C) participants. The FN facilitation at rest was increased in SCI (119.2% ± 9.3%) compared with control (111.0% ± 7.1%, p=0.007) participants. Thus, we tested the FN facilitation in a subgroup of SCI participants (n=10) by adjusting to magnitude of the intensity of the conditioning pulse to match the level of FN facilitation obtained in control participants (FN facilitation_adj_, see Materials and methods). We found that the FN facilitation_adj_ increased during 30% of MVC and rest in SCI participants (rest = 113.2% ± 6.2%, 30% of MVC = 120.2% ± 11.1%, p=0.01; Figure 5C–D) but to a lesser extent than controls.

### Correlation

**Figure 6.**
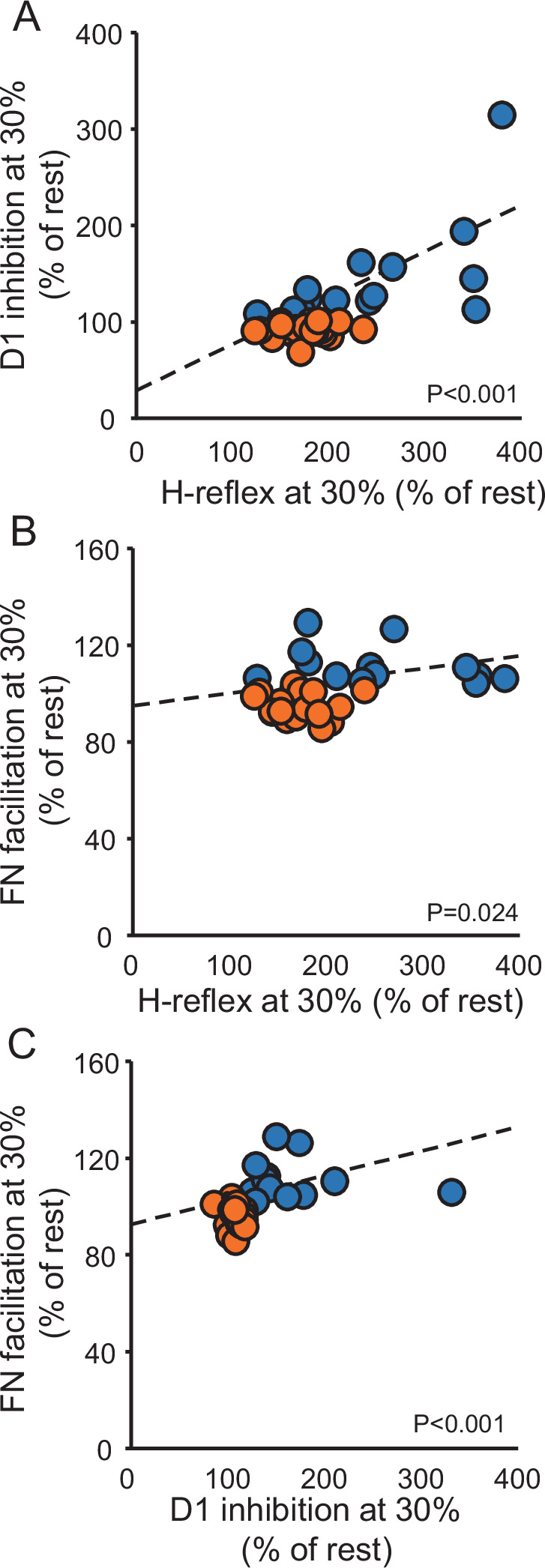
Correlation. Graphs show individual data from controls (blue circles) and SCI (orange circles) participants. The abscissa shows the size of the H-reflex during 30% of MVC expressed as a % of the H-reflex tested at rest (**A and B**) and the ordinate shows the D1 inhibition during 30% of MVC expressed as a % of the D1 inhibition tested at rest (**A**), the FN facilitation during 30% of MVC expressed as a % of the FN facilitation tested at rest (**B**). Data from individual subjects (**C**) showing the correlation between the FN facilitation during 30% of MVC expressed as a % of the FN facilitation tested at rest (C, abscissa) and the D1 inhibition during 30% of MVC expressed as a % of the D1 inhibition tested at rest (C, ordinate). Dashed lines represent the regression line of all the data points included in the plot. *p<0.05, Controls n = 14, SCI n = 20.

Figure 6 shows the correlation between the size of H-reflex, D1 inhibition, and FN facilitation during 30% of MVC compared with rest. We found that the H-reflex size positively correlated with the D1 inhibition (r=0.6, p<0.001; Figure 6A) and the FN facilitation (r=0.4, p=0.024; Figure 6B) during 30% of MVC compared with rest. The D1 inhibition positively correlated with the FN facilitation (r=0.7, p<0.001; Figure 6C) during 30% of MVC compared with rest. No correlation was found between the size of H-reflex and the D1 inhibition (r=0.2, p=0.2) and the size of H-reflex and the FN facilitation (r=0.1, p=0.6) during 30% of MVC compared with rest when adjusted data was used for the analysis.

## Discussion

Our electrophysiological data supports the hypothesis that during voluntary contraction the regulation of Ia afferent input to motor neurons is altered following SCI. The size of the H-reflex in the soleus muscle increases in controls and SCI participants during voluntary contraction but to a lesser extent in people with SCI. Two observations suggest that altered regulation of Ia afferent input from homonymous and heteronymous nerves during voluntary contraction might contribute to these results. First, the D1 inhibition was decreased in controls but it was still present in SCI participants during voluntary contraction compared with rest. Second, the FN facilitation was increased in controls but not in SCI participants during contraction compared with rest. Changes in the D1 inhibition and the FN facilitation correlated with changes in the H-reflex size during voluntary contraction. Together, these findings indicate that during voluntary contraction Ia afferent input from homonymous and heteronymous nerves exert a lesser facilitatory effect on motor neurons in humans with SCI compared with control subjects.

### Regulation of Ia afferent input after SCI

Although there have been multiple studies in animals and humans showing that sensory input conveying to motor neurons is altered following SCI (DAmico et al., 2014), the functional consequences of these changes remain largely unknown. Here, for the first time, we examined the regulation of Ia afferent input during voluntary contraction in humans with chronic incomplete SCI. Studies have used H-reflex conditioning paradigms in control humans to make inferences about the regulation of Ia afferent input (Hultborn et al., 1987a, Hultborn et al., 1987b; Meunier and Pierrot-Deseilligny, 1989; Nielsen and Kagamihara, 1993). For example, the depression of the soleus H-reflex evoked by CPN stimulation (referred here as D1 inhibition) is thought to be caused by presynaptic inhibition at the terminal of Ia afferents on soleus motor neurons (Mizuno et al., 1971) and the FN facilitation is thought to reflect the size of the monosynaptic EPSP in the soleus motor neurons evoked by activation of Ia afferents from the quadriceps muscle (Hultborn et al., 1987a). Both outcomes likely provide independent information about Ia afferent transmission that help to rule out changes in the recruitment gain of soleus motor neurons (Nielsen and Kagamihara, 1993). In the current study, the D1 inhibition was decreased during voluntary contraction in controls subjects but still present in SCI participants compared with rest. In addition, the FN facilitation was increased in control subjects during voluntary contraction but not in SCI participants when compared with rest. This is consistent with studies showing that, during a ramp-up and hold tonic contraction, in control subjects the FN facilitation decreased at the onset of a voluntary contraction (Hultborn et al., 1987a; Meunier and Pierrot-Deseilligny, 1989) and during a tonic contraction lasting for 1–2 min (Nielsen and Kagamihara, 1993) as performed in the present study. This is also consistent with findings in control subjects showing that vibratory inhibition of the soleus H-reflex decreases during tonic voluntary contraction (Iles and Roberts, 1987). These results were previously interpreted as a decrease in presynaptic inhibition during a voluntary contraction. For decades, it was thought that sensory regulation was accomplished in part through axoaxonic contacts at the terminal of Ia sensory axons through presynaptic inhibition (Frank and Fuortes, 1957; Eccles et al., 1961; Eccles et al., 1962; Rudomín et al., 1998). However, recent evidence in rats and control humans suggested that facilitation of Ia-mediated EPSPs in motor neurons likely occurs when axon nodes are depolarized from the activation of nodal GABA_A_ receptors (Hari et al., 2021; Metz et al., 2021). Thus, it is possible that the regulation of Ia afferent input tested in our study occurs not at the terminal of the Ia sensory fiber but at different sites within the Ia afferent fiber. It is also possible that the suppression of the H-reflex evoked by conditioning of a homonymous nerve is related, at least in part, to post-activation depression or any direct effect on the soleus motor neurons (Metz et al., 2022). Regardless of the site of Ia afferent regulation, together, these findings suggest that during voluntary contraction proprioceptive input from homonymous and heteronymous nerves exert a lesser facilitatory effect on motor neurons after SCI.

Both peripheral (Eccles et al., 1963; Jankowska et al., 2021) and central (Carpenter et al., 1963; Andersen et al., 1964; Fetz, 1968) mechanisms have been shown to contribute to regulate Ia sensory transmission. GABAergic neurons contributing to regulate Ia sensory transmission receive innervation from the brain and spinal cord (Rudomin and Schmidt, 1999). At rest, decreases in the D1 inhibition and increases in the FN facilitation in people with SCI compared with control participants could be related to decreased input from descending motor pathways. This is supported by multiple studies showing that activation of descending motor pathways, including the corticospinal pathway, is altered after the injury having a higher threshold resulting in the use of higher stimulus intensities in SCI compared with control participants (Bunday and Perez, 2012; Bunday et al., 2014; Cirillo et al., 2016). Then, how do we explain the facilitatory effect of Ia afferent input on motor neurons in control participants during voluntary contraction? A possibility is that this is, in part, related to descending inhibition of GABAergic interneurons, which overrides the suppression originated from peripheral sources during a voluntary contraction. Hence, in SCI participants, a lesser facilitatory effect of Ia afferent input on motor neurons might be present during voluntary contraction because of the abnormal and/or decreased contribution from descending pathways, which might not be strong enough to override peripheral sources. Participants in the present study had incomplete injuries and were able to perform voluntary contraction. Evidence showed that corticospinal excitability increases in controls and SCI participants during voluntary contraction but to a lesser extent in SCI participants (Davey et al., 1998; Bunday et al., 2014; Tazoe and Perez, 2021). Thus, a possibility is that the lesser facilitatory effect of Ia afferent input on motor neurons after SCI during voluntary contraction reflects altered contribution from descending motor pathways. Note that when the D1 inhibition and FN facilitation were tested at matching levels between groups, we observed a small but significant decrease in the D1 inhibition and increase in the FN facilitation during voluntary contraction, suggesting that to some extent similar mechanisms might contribute to the modulatinon of Ia afferent input in controls and SCI participants. This is also consistent with our results showing that H-reflex size increased during voluntary contraction in controls and SCI participants but to a lesser extent in people with SCI. Similarly, evidence showed that during voluntary contraction, motor neuron excitability (as measured by F waves) increases in people with SCI but to a lesser extent than in control participants (Vastano and Perez, 2020). Stretch reflexes (Woolacott and Burne, 2006) and H-reflexes Faist et al., 1994; Faist et al., 1996 have been reported to increase to a lesser extent or not at all during voluntary contraction in people with SCI compared with control subjects. Another important question is if changes in H-reflex size were related to changes in the D1 inhibition and FN facilitation. We did not find a correlation between these variables in each group. However, when we looked at the groups together, we found a strong positive correlation showing that increases in H-reflex size were associated with lesser D1 inhibition and larger FN facilitation, suggesting a relation between these variable in the overall population.

### Functional consequences

During a voluntary contraction, the ‘excitability’ of motor neurons increases. Thus, regulation of Ia afferent input to motor neurons can have implications for the generation of motor output. Indeed, evidence from animal studies and modelling analysis suggested that presynaptic regulation of spinal sensory feedback contributes to ensure smooth execution of movement (Fink et al., 2014) and motor stability (Stein and Oğuztöreli, 1976). In intact humans, lesser facilitatory effect of Ia afferent input on motor neurons have been related to the optimization needed to improve motor performance during motor skill learning (Perez et al., 2005). Thus, it is possibly that the lesser facilitatory effect of Ia afferent input on motor neurons during voluntary contraction in SCI compared with controls contributes to regulate the ongoing voluntary contraction. Because after SCI prolonged EPSPs are observed in motor neurons in response to even brief sensory stimulation (Norton et al., 2008), a lesser facilitatory effect of Ia afferent input on motor neurons at the spinal level in SCI participants might be beneficial to control small levels of ongoing voluntary contraction as the one performed in our study. It is unclear if this adaptation contributes to the lack of task-dependent modulation of the H-reflex observed in humans with SCI during the gait cycle (Yang et al., 1991; Phadke et al., 2010). H-reflex modulation differs during sitting, standing, and walking in humans with and without SCI (Hayashi et al., 1992; Phadke et al., 2010) requiring that future studies assess the impact of our results on gait-based and other conditions. In controls, the greater facilitatory effect of Ia afferent input onto motor neurons might be functionally appropriate during the tonic voluntary contraction that we tested, but this might also change to a different extent during performance of more skilled motor behaviors.

## Materials and methods

### Subjects

Twenty individuals with SCI (50.9±17.2 years, 2 women) and 20 control subjects (41.5±13.6 years, 6 women; F_1,38_=2.8, p=0.1) participated in the study. All subjects were provided written consent to experimental procedures, which were approved by the local ethics committee at Northwestern University (IRB protocol #STU00209996). Participants with SCI had a chronic injury (≥1 year) and were classified using the International Standards for Neurological Classification of Spinal Cord Injury (ISNCSCI) as having a C2-T10 SCI. Five out of the 20 subjects were categorized by the American Spinal Cord Injury Impairment Scale (AIS) as AIS C and the remaining 15 subjects were classified as AIS D. Nine individuals with SCI were currently taking anti-spastic medication (baclofen and/or tizanidine and/or gabapentin; Table 1) at the time of enrollment. These participants were asked to stop anti-spastic medication on the day of testing (at least 12 hr since last dosage). Spasticity was assessed using the Modified Ashworth Scale (MAS). All the SCI and controls participated in the H-reflex experiment, 6 of 20 controls were not able to come back to the lab for the conditioning H-reflex experiments. Sample size for the H-reflex testing was estimated using an effect size (η^2^p=0.3) calculated from the F-statistic (F_1,38_=15.6, p<0.001) for the significant GROUP ×CONTRACTION interaction that identified a lesser increase in H-reflex size in SCI compared to controls during voluntary contraction. With a power of 0.95 and α of 0.05, 36 participants were considered sufficient in a repeated measures ANOVA (G*Power 3.1.9.7). Additionally, sample size for the D1 inhibition and FN facilitation was estimated using an effect size (η^2^p=0.7 and η^2^p=0.6, respectively) calculated from the significant GROUP ×CONTRACTION interaction; with a power of 0.95 and α of 0.05, 14 and 18 participants (respectively) were considered sufficient in a repeated measures ANOVA (G*Power 3.1.9.7).

**Table 1.** Spinal cord injury (SCI) participants.

ID	Age (years)	Gender	AIS	MAS	Level	Time post injury (years)	Medication
1	61	F	D	1	T8	19	NO
2	48	M	D	3	C4	12	BAC
3	56	M	D	2	C4	9	GAB
4	41	M	D	2	C3	2	BAC,GAB
5	46	M	D	3	C5	8	BAC,GAB
6	30	M	C	1	C2	8	NO
7	37	M	D	4	C7	10	NO
8	71	M	D	3	T10	6	NO
9	73	M	C	1	C5	8	NO
10	38	M	D	2	C5	22	NO
11	33	M	D	2	C5	8	NO
12	64	M	C	2	C3	40	BAC,GAB
13	42	M	D	1	C5	11	NO
14	63	M	C	1	C5	6	TIZ
15	59	M	D	1	C7	11	BAC
16	28	M	D	4	C4	1	BAC
17	19	M	D	2	C5	2	NO
18	80	M	D	1	C4	9	NO
19	58	F	C	1	C3	5	GAB
20	72	M	D	0	C3	12	NO

M = male; F = female; AIS = American Spinal Injury Association impairment scale; MAS = Modified Ashworth Scale; BAC = baclofen; GAB = gabapentin; DIA = diazepam; TIZ = tizanidine.

### EMG recordings

EMG was recorded from the soleus and tibialis anterior muscle of the right side in control subjects and from the leg with the higher MAS score in individuals with SCI through bipolar surface electrodes (inter-electrode distance, 2 cm) placed over the belly of the muscle below the gastrocnemius muscles (AG-AgCl, 10 mm diameter). Note that the FN facilitation have been found to be different between participants with and without spasticity (Nielsen et al., 1995). Therefore, for standardization purposes, we recorded data from the more spastic side in each of the SCI participants. EMG signals were amplified, filtered (30–1000 Hz), and sampled at 2 kHz for both online detection (third-order Butterworth, 5–150 Hz band pass filtered and rectified) and offline analysis (CED 1401 with signal software, Cambridge Electronic design, Cambridge, UK).

### Experimental setup

During all testing procedures, subjects were seated comfortably in a custom armchair with both legs placed on a custom platform with the hip (~120°) and knee (~160°) flexed and the ankle restrained by straps in ~110° of plantarflexion. At the start of the experiment, participants performed three brief MVCs of 3–5 s into plantarflexion, separated by 30 s. The maximal mean EMG activity in the soleus muscle was measure over a period of 1 s on the rectified response generated during each MVC and the highest value of the three trials was used. The soleus H-reflex (Figure 1, see methods below) was tested at rest and during 30% of MVC (controls = 29.8% ± 7.1% of MVC and SCI = 33.2% ± 4.0% of MVC, p=0.2) into plantarflexion. Because MVCs were lower in SCI compared with control participants, we conducted additional experiments in control subjects (n=10) in which we matched the absolute EMG level exerted by SCI participants in all conditions. We also measured Ia afferent transmission (by testing the D1 inhibition and the FN facilitation; Figure 1) at rest and during 30% of MVC. Mean rectified EMG activity in the soleus muscle was shown online on a computer screen located in front of the participants by using Signal software to ensure that individuals were able to match EMG activity during all tasks.

### Soleus H-reflex

The soleus H-reflex was elicited by using electrical stimulation with the cathode positioned over the posterior tibial nerve in the popliteal fossa and the anode positioned above the patella using a constant-current stimulator (1 ms rectangular electrical stimulus, 0.25 Hz; model DS7A, Digitimer, Hertfordshire, UK). The reflex response was measured as the peak-to-peak amplitude of the non-rectified reflex response recorded from the soleus muscle. The stimulus intensity was increased in steps of 0.05 mA starting below H-reflex threshold and increasing up to supramaximal intensity to measure the M-max. To ensure that M-max values were reached, the stimulus intensity was increased until a plateau was observed in the M-max. The size of the H-reflex was kept at 50% of the H-max at rest and the same intensity was used during voluntary contraction. The magnitude of H-reflex during 30% of MVC was expressed as a % of the H-reflex at rest. Twenty reflexes were tested at rest and 20 reflexes were tested during 30% of MVC in a randomized order.

### D1 inhibition

The soleus H-reflex (control H-reflex) was conditioned by stimulation of the CPN (conditioned H-reflex). The CPN stimulation elicits a depression of the soleus H-reflex at conditioning-test interval of 8–20 ms (referred to as D1 inhibition; Mizuno et al., 1971). Consistent with previous results (Mizuno et al., 1971; Perez et al., 2005), we used a conditioning-test interval of 15 ms to assess the D1 inhibition at rest and during 30% of MVC in both controls (n=14) and SCI participants (n=20). The CPN was stimulated (1 ms rectangular electrical stimulus) through a bipolar bar electrode placed over the nerve distal to the neck of the fibula. The goal was to evoke a motor response in the tibialis anterior muscle without a motor response in the peroneal muscles. The intensity of the CPN stimulation was kept at 1.4×motor response threshold (MT) in the tibialis anterior muscle (Mizuno et al., 1971). The MT was defined as the minimal intensity needed to elicit 5 of 10 motor responses in the tibialis anterior muscle of 50 µV above the background. The stimulus evoking the H-reflex was adjusted to obtain the same size of the control H-reflex (50% of the H-max) both at rest and during voluntary contraction. We found that the magnitude of the D1 inhibition was decreased in SCI (81.2% ± 7.8%) compared with control (69.6% ± 15.4%, p=0.01) subjects at rest. Thus, in an additional control experiment, we tested the D1 inhibition in SCI participants (n=10) using a higher CPN stimulation at 1.5–2×MT while the control H-reflex was kept at 50% of the H-max to elicit a magnitude of D1 inhibition similar to controls (referred to as D1 inhibition adjusted, D1 inhibition_adj_). This subgroup of subjects were participants that were able to return to complete this testing. Fifteen control H-reflexes and 15 conditioned H-reflexes were tested at rest and during 30% of MVC in a randomized order.

### FN facilitation

The soleus H-reflex was tested with (conditioned H-reflex) and without (control H-reflex) stimulation of the FN. The FN elicits a facilitation of the soleus H-reflex (FN facilitation), which is thought to reflect the size of the monosynaptic EPSP in soleus motor neurons evoked by activation of Ia afferents from the quadriceps muscle, and changes in its size are considered to indicate changes in Ia afferent transmission (Hultborn et al., 1987b). Thus, both measurements, D1 inhibition and FN facilitation, provide independent information about Ia afferent transmission and help to rule out changes in the recruitment gain of soleus motor neurons (Nielsen and Kagamihara, 1993). The FN was stimulated through bipolar electrodes with the cathode positioned over the femoral triangle and the anode electrode positioned just below the gluteus maximus muscle. The intensity for stimulating the FN was 5×MT in the quadriceps muscle (Faist et al., 1994; Gracies et al., 1994). The onset of facilitation was taken to be the earliest conditioning-test interval at which the conditioned reflex was at least 5% larger than the control reflex to ensure that the conditioning-test interval reflects the size of the monosynaptic EPSP in the soleus motor neurons without contamination (Morita et al., 2001). Measurements were taken at 0.5–1 ms longer than this interval. The stimulus evoking the H-reflex was adjusted to obtain the same size of the control H-reflex (50% of the H-max) both at rest and during 30% of MVC. The facilitation induced by stimulating the FN has an onset at a conditioning-test interval between –7 and –8.5 ms (Nielsen and Kagamihara, 1993; Faist et al., 1994) (a negative value indicates that the control stimulus preceded the conditioning stimulus). In a control experiment, we tested conditioning-test intervals between –6.5 and –9.0 ms (a negative value indicates that the control stimulus preceded the conditioning stimulus) in controls (n=5) and SCI (n=5) and determined that in both groups the earliest onset of the FN was found at –7.5 ms (p=0.01). Thus, consistent with ours and previous results (Nielsen and Kagamihara, 1993; Faist et al., 1994), we used a conditioning-test interval of –8 ms to evaluate the FN facilitation at rest and during 30% of MVC in both controls (n=14) and SCI participants (n=20). The magnitude of the FN facilitation at rest is decreased in SCI compared with control subjects (Faist et al., 1994), therefore, in a control experiment we tested the FN facilitation in SCI participants (n=10) using lower FN stimulation at 2–4×MT (referred to as FN facilitation adjusted, FN facilitation_adj_) while the control H-reflex was kept at 50% of the H-max. The subgroup of subjects tested with adjusted stimulus intensities was the group of individuals that was able to return for this additional testing. Fifteen control H-reflexes and 15 conditioned H-reflexes were tested at rest and during 30% of MVC in a randomized order.

### Data analysis

Normal distribution was tested by the Shapiro-Wilk’s test and homogeneity of variances by the Levene’s test. Sphericity was tested using Mauchly’s test. When sphericity was not met, the Greenhouse-Geisser correction was used. Repeated measures ANOVA was used to examine the effect of GROUP (controls and SCI) and CONTRACTION (rest and 30% of MVC) on background EMG activity and the H-reflex size. Repeated measures ANOVA was also used to examine the effect of GROUP and CONTRACTION on the D1 inhibition and FN facilitation. Similar analysis was also used to examine the effect of CONTRACTION on the D1 inhibition_adj_ and the FN facilitation_adj_. One-way ANOVA was used to examine the effect of GROUP on MVCs, H-max, M-max, and the H-max/M-max ratio. Holm-Sidak post hoc analysis was used to test for mean pair wise comparisons. Spearman’s correlation coefficient was used to assess association between the size of H-reflex, D1 inhibition, FN facilitation during 30% of MVC compared with rest. Statistical analysis was conducted using SigmaPlot (Systat Software, Inc, San Jose, CA) and the significance was set at p<0.05. Group data is presented as means ± SDs. Effect sizes were reported as η^2^p.

## Data Availability

All data generated or analyzed during this study are included in the manuscript.

## Additional files

MDAR checklist
