# Supplementary material for: Altered regulation of Ia afferent input during voluntary contraction in humans with spinal cord injury
Source: eLife. 2022 Sep 7;11:e80089. doi: 10.7554/eLife.80089 (PMC9451536; doi:10.7554/eLife.80089)
Supplement: Peer review history

# Editor report: Editor's evaluation

**DOI:** 10.7554/eLife.80089.sa0

**Authors:** Christopher Cardozo

**Affiliations:** https://ror.org/04a9tmd77 Icahn School of Medicine at Mount Sinai United States

This paper will be of interest to basic and clinical neurophysiologists who are focused on understanding neural mechanisms that influence recovery following spinal cord injury (SCI). The work compares the afferent regulation of motor output to soleus muscle in controls and individuals with SCI. The results indicate differences between groups such that there is less facilitation in the SCI group during muscle contraction.

# Decision letter

**DOI:** 10.7554/eLife.80089.sa1

**Affiliations:** https://ror.org/04a9tmd77 Icahn School of Medicine at Mount Sinai United States; https://ror.org/04a9tmd77 Icahn School of Medicine at Mount Sinai United States; https://ror.org/02fa3aq29 McMaster University Canada

Our editorial process produces two outputs: (i) public reviews designed to be posted alongside the preprint for the benefit of readers; (ii) feedback on the manuscript for the authors, including requests for revisions, shown below. We also include an acceptance summary that explains what the editors found interesting or important about the work.

**Decision letter after peer review:**

Thank you for submitting your article "Altered Regulation of Ia Afferent Input during Voluntary Activity after Human Spinal Cord Injury" for consideration by *eLife*. Your article has been reviewed by 3 peer reviewers, including Christopher Cardozo as the Reviewing Editor and Reviewer #1, and the evaluation has been overseen by a Reviewing Editor and Mone Zaidi as the Senior Editor. The following individual involved in the review of your submission has agreed to reveal their identity: Aimee J Nelson (Reviewer #3).

The reviewers have discussed their reviews with one another, and the Reviewing Editor has drafted this to help you prepare a revised submission.

Essential revisions:

1. Please address the concerns of Reviewer 2 regarding the presentation of data in Figure 2.

2. Please carefully consider the remaining comments of the reviewers and revise the manuscript to address them or provide an explanation for why such revisions are not possible.

*Reviewer #1 (Recommendations for the authors):*

The manuscript would be significantly improved by addressing the following comments.

1. It sounds like the final N for controls was 14 subjects but this was not explicitly discussed. Were data for all 20 subjects used in some analyses but only 14 in others? How do the authors think the dropout of those subjects impacted statistical power? This is a minor point but one that should be considered since they provide an a priori sample size estimate of 36 subjects.

2. Given that *eLife* is not a specialty publication, I recommend the authors expand the introduction to give more information about where Ia fibers arise and what their function is. A bit more introductory background on what the neurophysiologic meaning is of what I understand to be the interaction between Ia signals arising in the femoral nerve with motor neurons for the soleus would also be helpful.

3. It would help the reader if the discussion were more clearly delineated when results of literature reporting neurophysiology for the able-bodied are discussed versus findings for persons with SCI.

4. Is there a teleological or gait-based way of thinking about the data that provides a model for understanding how gains or loss of effects of Ia inputs on H-reflexes are good or bad after SCI when muscles are already under load? Does this reasoning fit the new data presented?

5. Some interpretations of the correlations presented in Figure 6 should be added.

6. The key novel aspect as presented by the authors is performing H-reflex texts when a muscle is under load. A brief introduction of what is known of the neurophysiology driving the decision to perform these studies would help the reader understand why they are important. For example, how are inputs from tendons or joints participating in the physiology observed, realizing these were not explicitly measured or manipulated. The discussion could come back to this point more clearly.

7. I found that the writing is at times difficult to understand. Careful revision to improve clarity would help the reader.

*Reviewer #2 (Recommendations for the authors):*

What is the justification for recording from the leg with the higher MAS?

What does the dashed line represent in figure 6C? It doesn't align with the distribution of values.

The arbitrary use of ordinate range values makes interpretation more challenging. There is a pattern throughout the manuscript to manipulate the range to make differences seem larger than they are (Figure 2C, 4C/D, 6A-C). Additionally, the use of divergent ordinate ranges within the same figure makes it difficult for the reader to compare effects across groups (Figure 3D, 4C/D, 5C/D, 6). In the case of figure 3D, it undercuts your argument that H-reflex is attenuated in chronic SCI.

Why are the H-wave amplitudes for 30% MVC and the rest similar in Figures4 and 5 (panels A and B)? In Figure 3A, there is a clear difference in amplitude.

The yellow color for conditioned H-reflex in figures 4 and 5 is too difficult to see and is confusing with a similar color as the text "spinal cord injury".

In the Results section, only panel A of Figures 2 and 3 are referenced in the EMG and Soleus H-reflex paragraphs

Figure 2A, Change "Baseline" to "Rest" for consistency.

Figure 3A, Why is the gap larger between the Rest and 30% in the SCI group than in the control group?

In Figures4D and 5D, can you use color to indicate which individuals in the SCI group are also in the SCI adjusted group?

# Reply: Author response

**DOI:** 10.7554/eLife.80089.sa2

Essential revisions:

Reviewer #1 (Recommendations for the authors):

The manuscript would be significantly improved by addressing the following comments.

1. It sounds like the final N for controls was 14 subjects but this was not explicitly discussed. Were data for all 20 subjects used in some analyses but only 14 in others? How do the authors think the dropout of those subjects impacted statistical power? This is a minor point but one that should be considered since they provide an a priori sample size estimate of 36 subjects.

The reviewer raises a good point. We want to clarify that for the main experiment (H-reflex) we tested 20 control and 20 SCI participants. For measurements of D1 inhibition and FN facilitation, we tested 14 control and 20 SCI participants. Six control participants were not able to return for additional testing. Therefore, we recalculated the power for the D1 inhibition and FN facilitation. Note that our results indicate that we had sufficient power with the number of participants that were tested. The following information was added to the manuscript:

(page 15),

“Additionally, sample size for the D1 inhibition and FN facilitation was estimated using an effect size (η^2^p=0.7 and η^2^p=0.6, respectively) calculated from the significant GROUP × CONTRACTION interaction, with a power of 0.95 and α of 0.05, 14 and 18 participants (respectively) were considered sufficient in a repeated measures ANOVA (G*Power 3.1.9.7).”

2. Given that eLife is not a specialty publication, I recommend the authors expand the introduction to give more information about where Ia fibers arise and what their function is. A bit more introductory background on what the neurophysiologic meaning is of what I understand to be the interaction between Ia signals arising in the femoral nerve with motor neurons for the soleus would also be helpful.

As suggested the following information was added to the introduction:

(page 3),

“Primary afferent fibers (Ia) are rapidly conducting sensory fibers that originate from muscle spindle primary endings, which constantly monitors the rate at which a muscle stretch changes (Matthews, 1972). Ia afferent fibers bifurcate on entering the spinal cord and run several segments in both rostral and caudal directions in the dorsal columns and make contact with motor neurons (Pierrot-Deseilligny and Burke, 2012).”

3. It would help the reader if the discussion were more clearly delineated when results of literature reporting neurophysiology for the able-bodied are discussed versus findings for persons with SCI.

As suggested we now clearly defined in the discussion when results were related to control versus individuals with SCI.

4. Is there a teleological or gait-based way of thinking about the data that provides a model for understanding how gains or loss of effects of Ia inputs on H-reflexes are good or bad after SCI when muscles are already under load? Does this reasoning fit the new data presented?

The reviewer raises an interesting point. We propose that a lesser facilitatory effect of Ia afferent input on motor neurons at the spinal level in SCI participants might help to control small levels of ongoing voluntary activity as the one performed in our study. Because after SCI prolonged excitatory post-synaptic potentials are observed in motor neurons in response to even brief sensory stimulation (Norton et al., 2008), a lesser facilitatory effect of Ia afferent input on motor neurons at the spinal level in SCI participants might be beneficial to control small levels of ongoing voluntary activity as the one performed in our study. It is unclear if this adaptation contributes to the lack of task-dependent modulation of the H-reflex observed in humans with SCI during the gait cycle (Yang et al., 1991; Phadke et al., 2010). H-reflex modulation differs during sitting, standing, and walking in humans with and without SCI (Hayashi et al., 1992; Phadke et al., 2010) requiring that future studies assess the impact of our results on gait-based and other conditions. This information was added to the manuscript.

5. Some interpretations of the correlations presented in Figure 6 should be added.

We added the following information to discussion:

(page 13),

“Another important question is if changes in H-reflex size were related to changes in the D1 inhibition and FN facilitation. We did not find a correlation between these variables in each group. However, when we looked at the groups together, we found a strong positive correlation showing that increases in H-reflex size were associated with lesser D1 inhibition and larger FN facilitation, suggesting a relation between these variable in the overall population.”

6. The key novel aspect as presented by the authors is performing H-reflex texts when a muscle is under load. A brief introduction of what is known of the neurophysiology driving the decision to perform these studies would help the reader understand why they are important. For example, how are inputs from tendons or joints participating in the physiology observed, realizing these were not explicitly measured or manipulated. The discussion could come back to this point more clearly.

As suggested the following information was added to the introduction:

(page 5),

“The H-reflex, D1 inhibition, and FN facilitation were studied because these electrophysiological outcomes are modulated in a task-dependent manner during different motor behaviors providing a unique tool to assess the effect of sensory input onto motor neurons during voluntary activity (Pierrot-Deseilligny and Burke, 2012). The position of the ankle joint was maintained constant across conditions to standardize the possible effect of other inputs into the testing procedures.”

7. I found that the writing is at times difficult to understand. Careful revision to improve clarity would help the reader.

The manuscript has been proofread and carefully revised for clarity.

Reviewer #2 (Recommendations for the authors):

What is the justification for recording from the leg with the higher MAS?

Evidence has shown differences in the regulation of Ia afferent input from heteronymous nerves in people with SCI with and without spasticity (Nielsen et al., 1995). Therefore, for standardization purposes, we recorded data from the more spastic side in each of the SCI participants. This information was added to the manuscript.

What does the dashed line represent in figure 6C? It doesn't align with the distribution of values.

The dashed line in Figure 6 represent the regression line of all the data points included in the plot. This information was added to the figure legend.

The arbitrary use of ordinate range values makes interpretation more challenging. There is a pattern throughout the manuscript to manipulate the range to make differences seem larger than they are (Figure 2C, 4C/D, 6A-C). Additionally, the use of divergent ordinate ranges within the same figure makes it difficult for the reader to compare effects across groups (Figure 3D, 4C/D, 5C/D, 6). In the case of figure 3D, it undercuts your argument that H-reflex is attenuated in chronic SCI.

As suggested, we use the same range of values in the ordinate for all figures.

Why are the H-wave amplitudes for 30% MVC and the rest similar in Figures4 and 5 (panels A and B)? In Figure 3A, there is a clear difference in amplitude.

In Figures 4 and 5 (panels A and B), we showed the adjusted H-reflex. This is why the same size of the H-reflex is observed at rest and during 30% of MVC. This was clarified in the figure legend.

The yellow color for conditioned H-reflex in figures 4 and 5 is too difficult to see and is confusing with a similar color as the text "spinal cord injury".

We replaced the yellow color by gray in Figures 4 and 5.

In the Results section, only panel A of Figures 2 and 3 are referenced in the EMG and Soleus H-reflex paragraphs

We now referenced all panels in Figures 2 and 3.

Figure 2A, Change "Baseline" to "Rest" for consistency.

This was changed as suggested.

Figure 3A, Why is the gap larger between the Rest and 30% in the SCI group than in the control group?

We modified the figure and now the gap is consistent across groups.

In Figures4D and 5D, can you use color to indicate which individuals in the SCI group are also in the SCI adjusted group?

Now, individual data is color coded in Figures 4 and 5.
